# Down-Regulation of the Interferon Signaling Pathway in T Lymphocytes from Patients with Metastatic Melanoma

**DOI:** 10.1371/journal.pmed.0040176

**Published:** 2007-05-08

**Authors:** Rebecca J Critchley-Thorne, Ning Yan, Serban Nacu, Jeffrey Weber, Susan P Holmes, Peter P Lee

**Affiliations:** 1 Division of Hematology, Department of Medicine, Stanford University, Stanford, California, United States of America; 2 Department of Statistics, Stanford University, Stanford, California, United States of America; 3 Norris Comprehensive Cancer Center, University of Southern California, Los Angeles, California, United States of America; National Institutes of Health, United States of America

## Abstract

**Background:**

Dysfunction of the immune system has been documented in many types of cancers. The precise nature and molecular basis of immune dysfunction in the cancer state are not well defined.

**Methods and Findings:**

To gain insights into the molecular mechanisms of immune dysfunction in cancer, gene expression profiles of pure sorted peripheral blood lymphocytes from 12 patients with melanoma were compared to 12 healthy controls. Of 25 significantly altered genes in T cells and B cells from melanoma patients, 17 are interferon (IFN)-stimulated genes. These microarray findings were further confirmed by quantitative PCR and functional responses to IFNs. The median percentage of lymphocytes that phosphorylate STAT1 in response to interferon-α was significantly reduced (Δ = 16.8%; 95% confidence interval, 0.98% to 33.35%) in melanoma patients (*n* = 9) compared to healthy controls (*n* = 9) in Phosflow analysis. The Phosflow results also identified two subgroups of patients with melanoma: IFN-responsive (33%) and low-IFN-response (66%). The defect in IFN signaling in the melanoma patient group as a whole was partially overcome at the level of expression of IFN-stimulated genes by prolonged stimulation with the high concentration of IFN-α that is achievable only in IFN therapy used in melanoma. The lowest responders to IFN-α in the Phosflow assay also showed the lowest gene expression in response to IFN-α. Finally, T cells from low-IFN-response patients exhibited functional abnormalities, including decreased expression of activation markers CD69, CD25, and CD71; T_H_1 cytokines interleukin-2, IFN-γ, and tumor necrosis factor α, and reduced survival following stimulation with anti-CD3/CD28 antibodies compared to controls.

**Conclusions:**

Defects in interferon signaling represent novel, dominant mechanisms of immune dysfunction in cancer. These findings may be used to design therapies to counteract immune dysfunction in melanoma and to improve cancer immunotherapy.

## Abstract 2

Prompted by altered expression patterns of interferon-responsive genes in T and B cells, Peter Lee and colleagues find that lymphocytes from melanoma patients have defects in interferon signaling.

## Editors' Summary

**Background.:**

The immune system, in addition to fighting infections, provides one of the body's main defenses against cancer. During cancer development, normal cells acquire genetic changes that allow them to grow uncontrollably and to move around the body. Some of these changes alter the antigens (proteins recognized by the immune system) expressed on their surface. As a result, the immune system recognizes and eliminates the newly formed cancer cells. Tumors—large masses of cancer cells—occur when this immune surveillance fails. Some tumors, for example, hide from the immune system by altering the antigens they express. Others release factors that shut off the immune response. However, for many tumor types, it is not clear why immune surveillance fails during their development or why global immune suppression develops in most patients with advanced disease.

**Why Was This Study Done?:**

Scientists want to understand the molecular basis of immune dysfunction in patients with cancer because if they knew what had gone wrong with the immune system, they might be able to repair it. Also, there is considerable interest in immunotherapy for cancer—for example, treatment with interferons (proteins made by certain immune system cells that activate other immune cells and also kill tumor cells) and the development of vaccines to stimulate antitumor immune responses. So far, immunotherapy has not been very successful, probably because of the underlying dysfunction of the immune system in patients with cancer. Understanding this dysfunction might lead to improvements in immunotherapy, so in this study the researchers have investigated the molecular mechanism responsible for immune dysfunction in patients with metastatic melanoma, a deadly form of skin cancer.

**What Did the Researchers Do and Find?:**

The researchers purified lymphocytes (immune cells that are involved in antitumor responses) from the blood of patients with metastatic melanoma and healthy people and examined their patterns of gene expression using a technique called microarray expression profiling. CD8 T cells (which kill cells expressing foreign or altered antigens), CD4 T cells (which help other T and B lymphocytes do their jobs), and B cells (which make antibodies, proteins that recognize antigens and label cancer cells for destruction by the immune system) from patients with melanoma all expressed lower levels of 24 genes, and higher levels of one gene, than those from healthy individuals. 17 of these genes were interferon-stimulated genes, which encode proteins responsible for the effects of interferons. Therefore, the researchers checked the functional responses of patient and control lymphocytes to interferon. When interferon binds to lymphocytes, it triggers the addition of a phosphate group to the protein STAT1, which then induces changes in gene expression. STAT1 phosphorylation occurred in a lower percentage of patient lymphocytes than control lymphocytes in response to interferon-α (which is sometimes used to treat melanoma). The lymphocytes from one-third of the patients responded well to interferon-α, but those from the other patients showed little response. Furthermore, prolonged treatment with high concentrations of interferon-α partly overcame the defect in interferon signaling in patient lymphocytes. Finally, T cells from the patients failed to make the normal markers of immune cell activation or cytokines (proteins that mediate the killing of tumor cells) after exposure to activating stimuli and had reduced survival compared to control lymphocytes.

**What Do These Findings Mean?:**

These results indicate that for patients with metastatic melanoma defects in interferon signaling are an important contributor to immune dysfunction. They also show that T cells from patients with melanoma (particularly those who respond poorly to interferon-α) have functional abnormalities that make them less likely to recognize and deal with melanoma cells. These results need confirming in many more patients, but they nevertheless represent an important step toward understanding the immune dysfunction associated with advanced melanoma and possibly other tumors. In addition, the identification of two subgroups of patients—interferon responders and poor interferon responders—may explain why only some patients with melanoma benefit from treatment with interferon-α. It might, therefore, be possible to pre-select those who would benefit from this treatment (which has some serious side effects) by examining patient lymphocytes for interferon responsiveness.

**Additional Information.:**

Please access these Web sites via the online version of this summary at http://dx.doi.org/10.1371/journal.pmed.0040176.

- US National Cancer Institute information (in English and Spanish) for patients on the immune system and its involvement in cancer, and for patients and professionals on melanoma
- American Cancer Society information for patients on immunotherapy
- Cancer Research Institute (New York) web-based book on cancer and the immune system
- MedlinePlus encyclopedia pages on melanoma (in English and Spanish)
- Cancer Research UK patient information on melanoma, including information on immunotherapy

## Introduction

Cancer inhibits the immune system by various cellular and molecular mechanisms. Dysfunction of the immune system arises during the early stages of cancer and throughout progression to metastatic disease [1]. CD8 T lymphocytes specific for tumor-associated antigens (TAAs) are often present in the blood of cancer patients and accumulate in tumor-draining lymph nodes and in primary and metastatic tumor sites [2]. While TAA-specific CD8 T cells are elicited in the majority of patients receiving current peptide vaccines and other immunotherapies, they do not effectively control or eradicate tumors, and the presence or magnitude of these responses does not reliably correlate with clinical outcome [3]. Such cells may be specifically driven into apoptosis [4,5] or rendered nonresponsive (anergic) in vivo, preventing cytolytic responses against tumor cells and appropriate activation to stimuli. Indeed, dysfunction of TAA-specific CD8 T cells has been shown in melanoma and other cancers [2,6]. TAA-specific CD4 T cells have also been identified and are thought to help the function, persistence, and magnitude of antigen-specific CD8 T cell responses [7,8]. Current immunotherapeutic strategies are subject to the immunosuppressive effects of cancer and regulatory T cells, which likely contribute to their lack of success thus far [9–13]. The precise nature and molecular basis of immune dysfunction in the cancer state are not well defined. Elucidation of the mechanisms of immune dysfunction in cancer will allow rational design of strategies to reverse existing immune dysfunction and normalization of lymphocyte populations to improve the endogenous immune responses to cancer and to improve the efficacy of cancer immunotherapy.

We focused on the major lymphocyte populations that may be involved in antitumor responses and negatively impacted by cancer, specifically CD8 T cells, CD4 T cells, B cells, and CD56dim natural killer (NK) cells from patients with metastatic melanoma. Inhibition of these lymphocyte populations in cancer would allow tumor progression and hinder immunotherapeutic approaches. The gene expression profiles of these cells were studied using new generation DNA microarrays (Agilent Human 1A v2). While many DNA microarray studies use heterogeneous cell populations, such as tumor or peripheral blood mononuclear cell (PBMC) samples, our study utilized pure cell subsets, stringently sorted by flow cytometry, to enable precise analysis of the cells of interest. The aim of our study was to use the gene expression profiles of lymphocyte subsets from melanoma patients to elucidate aspects and mechanisms of immune dysfunction in cancer. These findings were confirmed by quantitative real-time PCR and further investigated via several novel assays including Phosflow analysis.

## Methods

### Patient and Healthy Donor Samples

PBMCs were obtained from patients with American Joint Committee on Cancer stage IV melanoma (University of Southern California Norris Cancer Center, Los Angeles, California), prior to biological or chemotherapy, with informed consent. All patients had visceral disease and all patients relapsed. PBMCs were also obtained from healthy donors that were age- and gender-matched to the patients (Stanford Blood Center, Stanford, California). PBMCs were cryopreserved in 90% NCS–10% DMSO. The experiments were approved by the Institutional Review Boards of University of Southern California Norris Cancer Center and Stanford University.

### Sorting of PBMCs into Lymphocyte Subsets

Cryopreserved PBMCs were thawed, extensively washed, and rested overnight in RPMI 1640 containing 10% FBS. PBMCs were washed and stained with antibodies to CD56, CD16, CD8, CD3, CD4, and CD19 (BD Biosciences, http://www.bdbiosciences.com; and Caltag, http://www.caltag.com) for 30 min. PBMCs were washed and resuspended in RPMI 50% FBS and kept on ice. PBMCs were sorted by flow cytometry using the BD FACSAria into CD8 T cells (CD3^+^ CD8^+^ CD4^−^ CD19^−^ CD56^−^ CD16^−^), CD4 T cells (CD3^+^ CD4^+^ CD8^−^ CD19^−^ CD56^−^ CD16^−^), B cells (CD19^+^ CD3^−^ CD8^−^ CD4^−^ CD16^−^ CD56^−^), and CD56dim NK cells (CD19^−^ CD3^−^ CD56dim CD16^+^) using the gates shown in Figure S1. All antibodies used in this study were fluorescently conjugated mouse anti-human monoclonal antibodies (BD Biosciences and Caltag). 200,000 cells were sorted into ice-cold RPMI with 50% FBS. The sorted cells were pelleted by centrifugation, homogenized in 1 ml of TRIzol (Invitrogen, http://www.invitrogen.com) plus 10 μg of linear acrylamide, and stored at −80 °C.

### Isolation and Checking of Total RNA

Total RNA was isolated from TRIzol homogenates according to the manufacturer's protocol and resuspended in RNase-free water. Genomic DNA was removed using the DNA-free kit (Ambion, http://www.ambion.com), according to the manufacturer's protocol. RNA quantity and quality were checked using the Nanodrop ND1000A spectrophotometer (Nanodrop Technologies, http://www.nanodrop.com) and the RNA 6000 Pico LabChip assay using the Agilent 2100 Bioanalyzer (Agilent Technologies, http://www.agilent.com).

### Total Lymphocyte Reference RNA for Two-Color DNA Microarrays

PBMCs were obtained from 20 healthy donors (ten male, ten female) ranging evenly in age from 25 to 65 years from the Stanford Blood Center. Granulocytes and monocytes were depleted from the PBMCs using RosetteSep depletion cocktails (Stemcell Technologies, http://www.stemcell.com). An aliquot of the purified lymphocytes was taken for antibody staining to determine purity. The cells were incubated with fluorescently conjugated monoclonal mouse anti-human antibodies to CD3, CD56, CD16, CD8, CD4, and CD19 for 30 min, washed, analyzed by flow cytometry (FACS Calibur, BD Biosciences) and were determined to be >99% pure lymphocytes. The remainder of the purified lymphocytes were homogenized in 10 ml of TRIzol plus 50 μg of linear acrylamide and stored at −80 °C. Total DNA-free RNA was isolated. 100 μg of total RNA from each of the 20 healthy donor total lymphocyte samples was pooled to create the total lymphocyte reference RNA.

### Amplification and Labeling of RNA

100 ng of total RNA from sorted cells or 1 μg of total lymphocyte reference RNA was amplified using the Amino Allyl MessageAmp II aRNA Amplification Kit (Ambion, http://www.ambion.com). The amplification procedure included incorporation of 5-(3-aminoallyl)-UTP (aaUTP) into aRNA during the in vitro transcription, to enable coupling to *N*-hydroxysuccinimidyl ester-reactive Cy dyes. aRNA yield and size were analyzed using the Nanodrop spectrophotometer and the RNA 6000 Nano LabChip assay. Amino-allyl-aRNA samples (2 μg each) were pooled in age- and gender-matched pairs, vacuum dried to completion, and resuspended in 8 μl of coupling buffer (Ambion). aRNA was labeled with 20,000 pmol of Cy Dye Post Labeling Reactive Dyes (Cy3 or Cy5) (Amersham Biosciences, http://www5.amershambiosciences.com) at room temperature for 30 min followed by addition of hydroxylamine to 0.18 M, for 15 min at room temperature, protected from light. Labeled aRNA was purified using the RNeasy MinElute Cleanup kit (Qiagen, http://www.qiagen.com). A_260_, A_550_, and A_650_ were measured, and number of dye molecules per 1,000 nucleotides was calculated using the formula: dye molecules/1,000 nt = (A_dye_/A_260_) × (9,101 cm^−1^ M^−1^ dye extinction coefficient^−1^) × 1,000. The labeling resulted in 30–60 dye molecules/1,000 nt.

### Hybridization and Processing of DNA Microarrays

750 ng of Cy Dye-labeled aRNA, 750 ng of differentially labeled total lymphocyte reference aRNA, and 50 μl of Agilent control targets were pooled and fragmented using the Agilent Fragmentation buffer for 30 min at 60 °C. Agilent hybridization buffer was added, and 490 μl of target solution was hybridized onto Agilent Human 1A Oligo Microarrays, v2 using the SureHyb (Agilent) assembly and incubated at 60 °C at 4 rpm for 17 h. The arrays were washed and dried according to the Agilent SSPE wash protocol, scanned using the Agilent Microarray Scanner and the data was extracted using the Agilent Feature Extraction Software v7.1.

### Analysis of Microarray Data

The open source R software package (http://www.r-project.org) and tools from the BioConductor project (http://www.bioconductor.org) were used for processing and analysis of the microarray data. The raw dataset contained 48 arrays: six healthy and six melanoma arrays for each of the four cell types. Two of the arrays in the data had quality problems due to background noise and were excluded from subsequent analysis. The manufacturer-designed control features and features that were saturated on at least one array were removed. For each array the variance-stabilizing normalization function was applied to the mean foreground Cy5 and Cy3 intensities, resulting in a “generalized log ratio” value for each feature, followed by quantile normalization across the arrays. The features were mapped to Entrez Gene IDs using the Agilent Human 1A (V2) Annotation Data (hgug4110b) package provided by BioConductor. Values from duplicate spots for each Entrez Gene ID were summarized using the median. The resulting data matrix contained 46 log ratio values for 16,476 genes. The array data from each cell type were compared separately and in various combinations in melanoma versus healthy samples. For each combination of two or more cell types a lower threshold of 0.01 was used for the *p*-value from an F-test comparing the cell types for each gene, such that only genes expressed at similar levels across involved cell types were included in the comparison. A subsequent nonspecific filtering selected the top 1,000 genes ranked by IQR across all relevant arrays. For each selected gene a permutation-based unadjusted *p*-value estimate [14] was extracted from the rawp component in the output of the maxT function in Bioconductor's multtest package. The *p*-values were then adjusted for multiple comparisons using the false discovery rate–controlling method [15].

### Real-Time Quantitative PCR

Unamplified total RNA was reverse transcribed using Sensiscript (samples <50ng total RNA) or Omniscript (samples 0.05–2 μg total RNA) reverse transcriptases (Qiagen) with 10 μM anchored Oligo(dT)_23_ primer and 10 μM random nonamers in a total volume of 20 μl at 37 °C for 1 h according to the manufacturer's protocol. 1 μl of cDNA was used as a template in qPCR reactions in duplicate or triplicate using SYBR Green Real Time PCR mix (Qiagen) and 300 nM specific primers. The PCR primers (Table S1) were designed using the Oligo 6.86 software (Molecular Biology Insights, http://www.oligo.net) to have equivalent melting temperatures. At least one primer of each pair spanned an exon–intron junction. The primers were synthesized by Elim Biopharmaceuticals (http://www.elimbio.com). Quantitative PCR analysis was performed using the iCycler iQ Real-Time Detection System (Bio-Rad Laboratories, http://www.bio-rad.com) with the following conditions: 15 min at 95 °C initial denaturation, 40 cycles of 30 s at 95 °C, 30 s at 56 °C, 30 s at 72 °C, followed by melting analysis.

### Analysis of Real-Time Quantitative PCR Data

The expression of each gene was normalized to the housekeeping gene *ubiquitin C (UBC)*. Two-sided Wilcoxon rank sum tests were used to compare the healthy and melanoma populations.

### Interferon Stimulation and Detection of STAT1-pY701

PBMCs were stained with antibodies to CD8, CD4, and CD19 for 30 min, washed and resuspended to 5 × 10^6^ cells per test in RPMI 5% human serum and rested at 37 °C 7% CO_2_ for 2 h. IFN-α, IFN-β, or IFN-γ (NIAID Reference Reagent Repository, http://www.kamtekinc.com/niaid.php; and R&D Systems, http://www.rndsystems.com) were added to a final concentration of 1,000 IU/ml and cells were incubated at 37 °C and 7% CO_2_ for 15 min. The cells were fixed by addition of Cytofix buffer (BD Biosciences) for 15 min at 37 °C and 7% CO_2_, and permeabilized in Perm Buffer III (BD Biosciences) for 30 min on ice. The cells were washed in stain buffer (1× PBS, 2% NCS, 0.09% sodium azide) and resuspended to 20 μl. The cells were stained with 5 μl of anti-STAT1-pY701 Alexa Fluor 647 at room temperature for 30 min, washed twice in stain buffer, and analyzed using the FACS Aria.

### Anti-CD3 Anti-CD28 Stimulation of T Cells and Measurement of T Cell Activation

Lymphocytes were stimulated with Dynabeads CD3/CD28 T Cell Expander beads (Invitrogen) at a bead:T cell ratio of 1:10 for measurement of CD69, CD25, and CD71, and at 1:1 for intracellular cytokine staining, at 37 °C and 7% CO_2_ for 24 h. For measurement of CD69, CD25, and CD71, cells were stained with antibodies to CD8, CD4, CD45RA, CD27, CD19, CD69, CD25, and CD71 for 30 min. For intracellular cytokine staining, 5 μg/ml brefeldin A was added to cells for the final 22 h of incubation. Cells were stained with antibodies to CD8, CD4, and CD19, and then fixed in 2% paraformaldehyde, washed, and permeabilized in Hank's balanced salt solution containing 0.1% saponin and 0.01 M Hepes. Cells were stained with antibodies to interleukin (IL)-2, TNF-α, and IFN-γ. Cells were washed and analyzed using the FACS Aria.

### Measurement of T Cell Survival

Cell survival was measured using the Annexin V:PE Apoptosis Detection Kit (BD Biosciences). Cells were stained with antibodies to CD8, CD4, CD45RA, CD27, and CD19 for 30 min, and washed in Annexin V binding buffer. Annexin V-PE and 7-aminoactinomycin D (7-AAD) were added to cells for 15 min at room temperature. Cells were diluted in Annexin V binding buffer and analyzed using the FACS Aria.

### Analysis of Flow Cytometry Data

FCS files were analyzed using Flowjo 8.2 (Treestar, http://www.treestar.com). The percentage of positive cells was calculated using the Super Enhanced D-max Subtraction method in Flowjo's Population Comparison platform. Probability Binning (Chi[T]) in the Population Comparison platform was used to quantitatively detect differences between IFN-α-stimulated cells versus unstimulated cells. One-sided Wilcoxon rank sum tests were used to make comparisons between melanoma and healthy Phosflow datasets. A binomial test was used to evaluate the systematic trend observed in a collection of comparisons on independent parameters measured in the FACS-based functional assays.

## Results

### Target Preparation and Microarray Quality Controls

CD8 T cells, CD4 T cells, B cells, and CD56dim NK cells were sorted to >99% purity from PBMCs from 12 patients (six male and six female) with metastatic melanoma and age- and gender-matched healthy controls (Figure S1). The mean and standard deviation (in parentheses) of the ages of the melanoma patients (*n* = 12) and healthy donors (*n* = 12) were 59.8 (9.6) y and 59.3 (9.2) y, respectively. The percentages of each cell type and the naïve, effector, and memory subsets were measured prior to sorting; there were no significant differences in these percentages in PBMCs from melanoma patients versus healthy controls. Total RNA isolated from the sorted cells was small (∼200 ng); therefore, amplification of polyadenylated RNA was carried out to produce sufficient target material for array hybridization. Amplification has been shown to improve the reliability of microarray data, and the bias introduced is minimal [16]. A total lymphocyte reference RNA was created from the total peripheral lymphocyte fraction from 20 healthy donors of a wide variety of ages. This reference is much closer to the samples than the standard reference for microarray experiments, the Universal Human Reference RNA (Stratagene, http://www.stratagene.com), thus maximized our ability to resolve subtle but statistically significant gene expression differences between melanoma lymphocyte subsets and healthy controls.

The reliability of the microarray data was evaluated by quality control experiments. Dye swap and self–self hybridizations showed correlations of 0.988 and 0.992, respectively, indicating very high consistency between the two labeling colors (Figure S2A and S2B). Replicate arrays within and between batches were included to measure the reproducibility of the hybridizations. The array-to-array correlation for pairs of replicate arrays within a batch was 0.987 (Figure S2C), and the batch-to-batch correlation ranged from 0.971 to 0.992**.** Prior to labeling and hybridization, aRNA samples were pooled into age- and gender-matched pairs, i.e., the 12 melanoma patient samples were hybridized as six pairs of samples, and each pair was gender-matched and closely age-matched. This pooling strategy has been shown to reduce the variation and noise in microarray data and to increase statistical power [17,18]. We chose this strategy for our experiments to further improve the sensitivity to detect subtle differences in gene expression between the patients and healthy donors.

### Gene Expression Changes in T and B, but Not NK Cells from Melanoma Patients versus Healthy Controls

The open-source R software package (http://www.r-project.org) and tools from BioConductor (http://www.bioconductor.org) were used for processing the microarray data as described in Methods. For CD8 T cells, CD4 T cells, and B cells, a similar set of genes with common regulation by IFNs showed reduced expression in patients with melanoma versus healthy controls. As such, data from these three cell types were combined to increase the statistical power of the analysis. Of the top 25 genes ranked by adjusted *p*-value, 17 are regulated by IFNs (Table 1). The products of IFN-stimulated genes (ISGs) are responsible for the antiviral, antiproliferative, and immunomodulatory effects of IFNs.

**Table 1:**
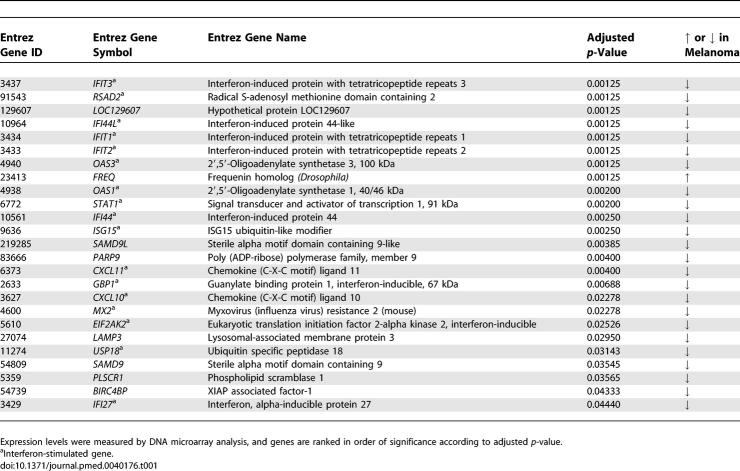
Genes Showing Differential Expression between Patients with Melanoma and Healthy Controls in Combined Microarray Data from CD8 T Cells, CD4 T Cells, and B Cells

Of 1,000 genes that were used as input into the multiple testing procedure, 25 were ISGs; 17 of these 25 genes have an adjusted *p*-value < 0.05, in contrast to eight of 975 non-IFN-stimulated genes that have an adjusted *p*-value < 0.05. A hypergeometric test was used to evaluate this over-representation of ISGs in the group of genes discriminating melanoma lymphocytes from healthy controls. The resulting *p*-value was less than 10^−27^, strongly indicating the reduced expression of ISGs as a highly significant difference between lymphocytes from patients with melanoma and those from healthy controls. An extensive study of how the differential expressions in T cells and B cells are related was done through a gene interaction program (Nacu et al., unpublished technical report, 2006, Department of Statistics, Stanford University).

**Figure 1:**
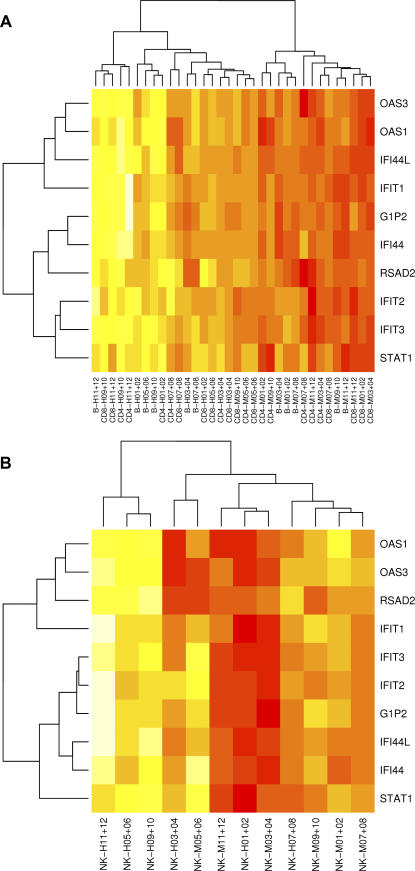
Hierarchical Clustering of the Microarray Data Using ISGs Hierarchical clustering of the microarray data was performed using the ten ISGs with lowest adjusted *p*-values in combined data from B cells, CD8 T cells, and CD4 T cells (A) and CD56dim NK cells (B) from patients with melanoma versus healthy controls. White indicates highest expression, red indicates lowest, and yellow/orange indicates intermediary expression in melanoma versus healthy control.

Hierarchical clustering of the microarray data was performed using the heatmap function in R for the top ten ISGs ranked by adjusted *p*-value for CD8 T cells, CD4 T cells, and B cells. This clustering separated the samples into two main groups, one of patients with melanoma with low expression of this set of ISGs and another of mainly healthy donors with higher expression (Figures 1A and S3A–S3C). Three arrays from two pairs of patients with melanoma fell into an intermediary subset containing healthy samples with expression intermediate between the low ISG-expressing melanoma and the high ISG-expressing healthy samples (Figure 1A).

Interestingly, no significant alterations in gene expression were detected between NK cells from melanoma patients versus healthy controls. The set of ISGs did not discriminate between the NK cells from melanoma and healthy samples (Figure 1B). Moreover, the top ranked genes for NK cells were also not able to discriminate between the two groups (Figure S3D), further confirming that the gene expression profiles of NK cells are not altered in melanoma patients.

### Validation of Microarray Data by Quantitative PCR

The altered expression of ISGs in T and B cells from patients with melanoma observed in the microarray experiments was validated by real-time quantitative PCR (qPCR) analysis of these genes using unamplified RNA reserved from the original, sorted samples prior to microarray analysis. The qPCR analysis showed reduced expression of ISGs in CD8 T cells, CD4 T cells, and B cells from patients with melanoma versus healthy controls, validating the microarray data (*p* < 0.05 for each gene, Table 2). The mean fold change in expression of these genes between the melanoma and healthy samples ranged from 1.544× for *HERC5* to 3.005× for *IFI44* (Table 2). *HERC5* gene regulation is not well understood, but expression of *HERC5* is induced in response to IFN-α (unpublished data). Two other non-IFN-regulated genes were also found to be significantly altered in expression in lymphocytes from patients with melanoma compared to healthy controls: *LAMP3* expression was reduced and *FREQ* expression was increased (Table 1). These expression changes were also validated by qPCR, with *p*-values of 0.006 and 0.054 for *LAMP3* and *FREQ,* respectively. A set of the top-ranking genes for NK cells from patients with melanoma and ISGs were chosen for analysis by qPCR. The expression of these two sets of genes in NK cells was not significantly different between patients with melanoma and healthy controls (Table S2).

**Table 2:**
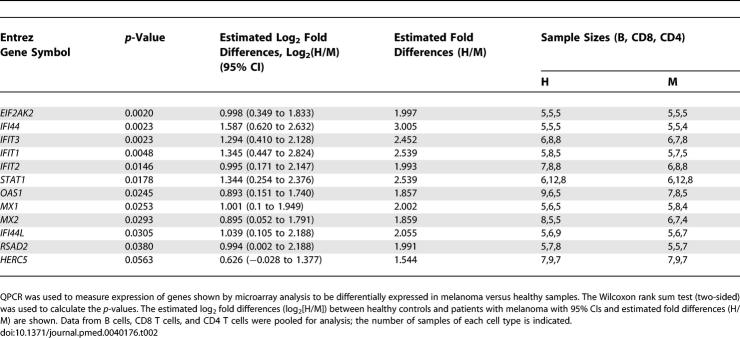
Real-Time Quantitative PCR Analysis of Genes Differentially Expressed in CD8 T Cells, CD4 T Cells, and B Cells from Patients with Melanoma versus Healthy Controls

### STAT1-Tyrosine Phosphorylation in Lymphocytes

**Figure 2:**
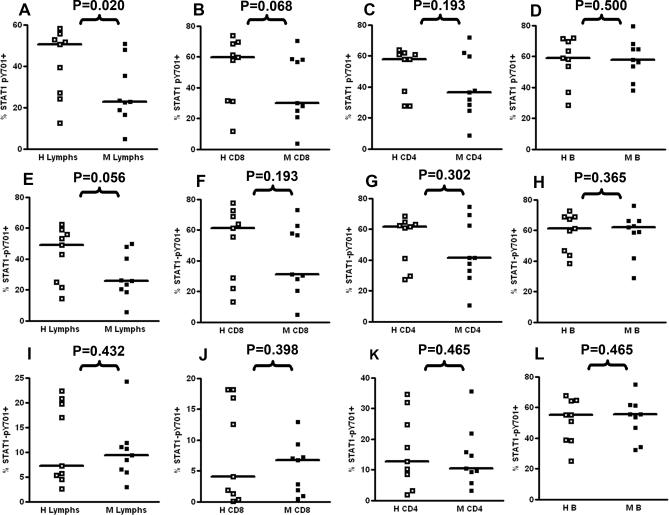
Percentage of STAT1-pY701-Positive Cells in Lymphocytes from Patients with Melanoma and Healthy Controls in Response to IFN Stimulation PBMCs from patients with melanoma and healthy controls were stimulated with 1,000 IU/ml IFN-α, IFN-β, or IFN-γ. The percentage of STAT1-pY701-positive cells was measured by Phosflow. *p*-Values were calculated using the Wilcoxon rank sum test (one-sided). Medians are indicated by the bar in each scatter column. H, healthy; M, melanoma. (A–D) IFN-α-stimulated PBMCs. Lymphocytes (A); CD8 T cells (B); CD4 T cells (C); B cells (D). (E–H) IFN-β-stimulated PBMCs. Lymphocytes (E); CD8 T cells (F); (G) CD4 T cells (G); B cells (H). (I–L) IFN-γ-stimulated PBMCs. Lymphocytes (I); CD8 T cells (J); CD4 T cells (K); B cells (L).

The reduced expression of *STAT1* and ISGs in T and B cells from patients with melanoma indicates a perturbation in IFN signaling in the immune system of these patients. To test this hypothesis, the functional response of lymphocytes to IFN stimulation was assessed by measurement of STAT1 phosphorylation, an essential event in signal transduction by IFNs. PBMCs from nine patients with melanoma and nine healthy controls were stimulated with 1,000 IU/ml IFN-α, IFN-β, or IFN-γ (or left unstimulated), and phosphorylation of STAT1 at tyrosine 701 was measured using Phosflow (BD Biosciences). The Phosflow method has greater sensitivity and is more quantitative than other methods, and it allows simultaneous analysis of multiple populations in small clinical samples. The concentration of 1,000 IU/ml was chosen for two reasons: (i) this concentration produced maximal induction of STAT1-pY701 for detection by the assay in titration experiments, and (ii) this is approximately the serum concentration of IFN-α2b reached in humans after intravenous infusion of IFN-α2b in the high-dose treatment regimen of 2 × 10^7^ IU/m^2^ (unpublished data). The median percentage of phosphorylated STAT1-positive lymphocytes induced by IFN-α stimulation was significantly reduced (Δ = 16.28%; 95% CI, 0.98 to 33.35, Figure 2A) in the patients with melanoma (*n* = 9) compared to the healthy controls (*n* = 9). Within the lymphocyte population, a reduction was observed in CD8 (Δ = 10.18%) and CD4 T cell (Δ = 8.71%) subsets, but not in B cells (Δ = 0.33%) (Figure 2B–2D). A similar pattern of reduction in the median percentage of STAT1-pY701-positive lymphocytes was observed with IFN-β stimulation (Figure 2E–2H). In response to IFN-γ stimulation, no significant difference was observed in phosphorylation of STAT1 in melanoma patient samples compared to controls (Figure 2I–2L). These results also indicate that there are two groups of patients: high (3/9) and low (6/9) responders to IFN-α.

The Probability Binning (Chi[T]) function in Flowjo [19] was used to compare the distributions of STAT1-pY701-stained cells in IFN-α-stimulated lymphocytes to corresponding unstimulated controls. Melanoma samples had a lower mean Chi^2^(T) value (3,830 ± 978.5) than the healthy (6,398 ± 1,453) for STAT1-pY701, further indicating that the induction of STAT1-pY701 by IFN-α stimulation was lower in melanoma compared to the healthy samples.

**Figure 3:**
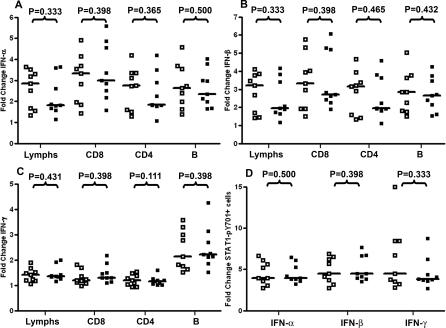
Fold Change in Mean Fluorescence Intensity of STAT1-pY701-Alexa Fluor 647 Staining in Lymphocytes from Patients with Melanoma and Healthy Controls in Response to IFN Stimulation The mean fluorescence intensity (MFI) of STAT1-pY701-Alexa Fluor 647 staining was measured by Phosflow in IFN-stimulated (1,000 IU/ml) and unstimulated cells. Total fold change = MFI stimulated cells/MFI-unstimulated cells; positive cell fold change = MFI STAT1-pY701-positive cells/MFI-unstimulated cells. *p*-Values were calculated using the Wilcoxon rank sum test (one-sided). Medians are indicated by the bar in each scatter column. Healthy (□; *n* = 9); melanoma (▪; *n* = 9). (A) IFN-α-stimulated cells. (B) IFN-β-stimulated cells. (C) IFN-γ-stimulated cells. (D) Positive cell fold change in lymphocytes.

Two comparisons were made of the fold change in mean fluorescence intensity of STAT1-pY701 staining in stimulated versus unstimulated cells: the total fold change in stimulated versus unstimulated cells, and the fold change in STAT1-pY701-positive cells versus unstimulated cells. The total fold change in STAT1-pY701 staining in IFN-α and IFN-β, but not IFN-γ, in stimulated versus unstimulated cells was systematically lower in CD8 (healthy [H], 2.76×; melanoma [M], 1.84×) and CD4 T cell (H, 3.32×; M, 2.9×) subsets and in the lymphocyte population as a whole (H, 2.84×; M, 1.81×) (Figure 3A–3C), supporting the results demonstrating reduced response of lymphocytes to type I IFNs. The fold change in mean fluorescence intensity of STAT1-pY701-positive cells versus unstimulated cells was not significantly different between healthy (3.95×) and melanoma (3.95×) lymphocytes (Figure 3D). These results indicate that those cells able to respond to IFN in the melanoma samples do so to a level similar to healthy lymphocytes. In the melanoma group, the fold change in STAT1-pY701-positive cells was significantly correlated with the percentage of STAT1-pY701-positive cells in response to IFN-α (*r* = 0.8841, *p* = 0.0016). In contrast, there was a lower and non-significant correlation (*r* = 0.5785, *p* = 0.1027) in the healthy group. This result indicates that the lower percentage of cells responding to IFN-α in the melanoma patient group is associated with a lower magnitude of response to IFN-α in these cell populations. Overall, these functional assays indicate a defect specifically in type I IFN signaling in T cells from patients with melanoma.

### Expression of ISGs in Lymphocytes upon IFN Stimulation

**Figure 4:**
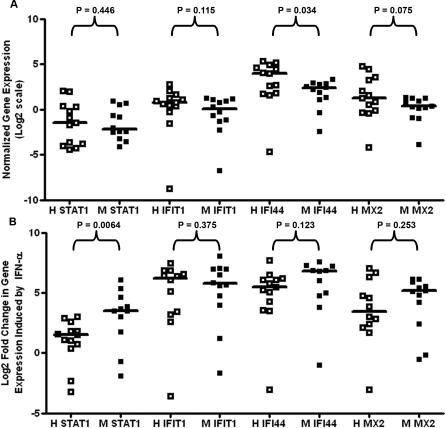
Expression of ISGs in Lymphocytes from Patients with Melanoma and Healthy Donors Stimulated with IFN-α Lymphocytes from patients with melanoma and healthy controls were stimulated with 1,000 IU/ml for 14 h. The expression of *STAT1, IFIT1, IFI44,* and *MX2* were measured by qPCR. Data are shown on a log_2_ scale. Medians are indicated by the bar in each scatter column. *p*-Values are from one-sided Wilcoxon rank sum test. H, healthy; M, melanoma. (A) Gene expression in IFN-α-stimulated cells, sample sizes were *n* = 13 (H) and *n* = 12 (M) for each gene. (B) Fold change in gene expression in IFN-α–stimulated cells versus unstimulated control cells. STAT1: *n* = 13 (H), *n* = 11 (M); IFIT1: *n* = 12 (H), *n* = 12 (M); IFI44: *n* = 13 (H), *n* = 11 (M); MX2: *n* = 12 (H), *n* = 12 (M).

The reduced phosphorylation of STAT1 may impair the ability of cells to induce downstream expression of ISGs. To test this hypothesis, lymphocytes from patients with melanoma and healthy controls were stimulated with 1,000 IU/ml IFN-α or unstimulated for 14 h, after which the cells were homogenized in TRIzol for RNA isolation, followed by cDNA synthesis. Real-time qPCR was performed for four ISGs shown to be significantly down-regulated in the microarray data: *STAT1, IFIT1, IFI44,* and *MX2*. The expression level of these genes in IFN- α~-stimulated cells was lower in the melanoma group compared to the healthy group, although the difference in expression was not statistically significant for three of the four genes (Figure 4A). This indicates that prolonged exposure to high-dose IFN could partially overcome the defect in IFN signaling in lymphocytes from patients with melanoma. The fold change in expression of *STAT1, IFI44,* and *MX2* in stimulated versus unstimulated cells (calculated using the Pfaffl method [20]) was higher in the melanoma patient samples (Figure 4B). Despite the higher median fold change in these patients, the level of expression of these ISGs did not reach that observed in the healthy samples (Figure 4A). The patients with the lowest basal expression of these genes had the lowest fold change in response to IFN-α; e.g., melanoma patients #2 and #11 are two of the lowest-ranking in basal expression of *STAT1, IFIT1, IFI44,* and *MX2* and showed the lowest fold changes for these genes, and the lowest response to IFN-α in the Phosflow assay out of the group of patients with melanoma, further indicating that some of these patients are IFN-low-responders. Patients with higher basal expression of these genes similar to the healthy group had higher fold changes in expression of these ISGs, indicating that other patients are IFN responsive. The difference in patient responses may explain why some patients with melanoma respond to high-dose IFN-α2b therapy while others do not.

### T Cell Expression of Activation Markers and Survival Following Stimulation

**Figure 5:**
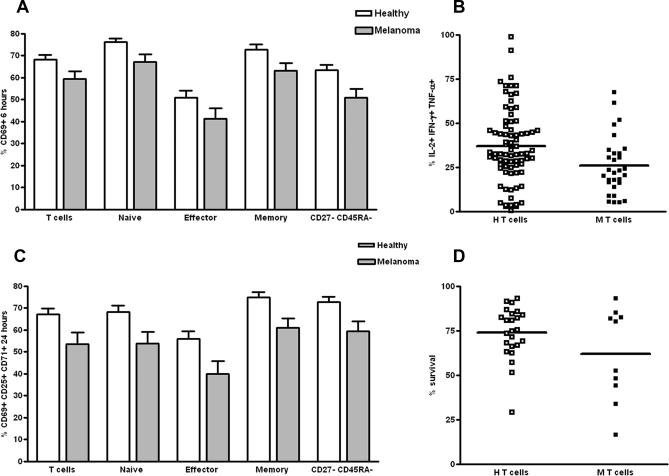
Activation and Survival of CD8 and CD4 T Cells in Response to Activating Stimulus Lymphocytes from patients with IFN-nonresponsive melanoma and healthy donors were stimulated with beads coated with anti-CD3 and anti-CD28 antibodies. (A) Percentage of CD8 and CD4 T cells expressing CD69 at 6 h following stimulation (mean and standard error). (B) Percentage of CD8 and CD4 T cells expressing CD69, CD25, and CD71 (data combined) at 24 h following stimulation (mean and standard error). (C) Percentage of CD8 and CD4 T cells expressing IL-2, TNF-α, and IFN-γ (data combined) at 24 h following stimulation. (D) Percentage of surviving (Annexin V-negative 7-AAD-negative) cells at 4 d following stimulation. Means are indicated by the bar in scatter columns.

Type I IFNs have both direct and indirect roles in supporting full activation and survival of T cells [21]. The impaired response to type I IFN may negatively impact the function of T cells in melanoma. To determine the functional status of T cells from the patients with melanoma, lymphocytes were stimulated with beads coated with anti-CD3 and anti-CD28 antibodies. T cell responses were assayed by measurement of surface markers of activation and cytokine production. At 6 h poststimulation, expression of CD69 was lower in the IFN-low-response patients (*n* = 5) compared to the healthy controls (*n* = 10) in each of the naïve, effector and memory, and CD27− CD45RA− subsets of CD8 and CD4 T cells (Figures 5A and S4). Correlations were made to determine whether the low response to IFN in the Phosflow assay was associated with the reduced expression of CD69 in lymphocytes from patients with melanoma following CD3/CD28 stimulation. The expression of CD69 was significantly correlated with the percentage of STAT1-pY701-positive lymphocytes following IFN-α stimulation (*r* = 0.9829, *p* = 0.0027, Figure S5) in the IFN-low-response patients. In contrast, the melanoma group as a whole showed lower correlation between the percentage of STAT1-pY701-positive lymphocytes following IFN-α stimulation and the expression of CD69 (*r* = 0.6786, *p* = 0.0643), while in the healthy group there was no correlation at all in this respect (*r* = −0.0044, *p* = 0.9909, Figure S5). These correlation results indicate an association between low response to IFN and reduced expression of CD69 by lymphocytes from IFN-low-response patients and a more pronounced functional defect in these patients compared to the melanoma group as a whole.

At 24 h poststimulation, expression of three early and late surface markers of activation—CD69, CD25, and CD71—was systematically reduced in the IFN-low-response patients' (*n* = 3) compared to the healthy controls' (*n* = 13) CD8 and CD4 T cells (Figures 5B and S6–S8). The expression of T_H_1-type cytokines IL-2, TNF-α, and IFN-γ was also reduced in T cells from these patients (*n* = 5) versus healthy controls (*n* = 13) in response to stimulation (Figures 5C and S9). There was a higher correlation between the percentage of stimulated CD8 or CD4 T cells expressing IL-2 with the percentage of STAT1-pY701-positive CD8 or CD4 T cells following IFN-α stimulation in the IFN-low-response group of patients (CD8, *r* = 0.9065; CD4, *r* = 0.4670, respectively) compared to the melanoma group as a whole (CD8, *r* = 0.1086; CD4, *r* = 0.01028) or the healthy donors (CD8, *r* = −0.35580; CD4, *r* = 0.1242), indicating that there is a close link between impaired response to IFN and low expression of IL-2 in the IFN-low-response group of patients with melanoma, and that this group of patients has a more pronounced functional defect than the melanoma group as a whole. The survival of T cells following stimulation was also measured. The survival percentage (Annexin V-negative 7-AAD-negative cells) was reduced in CD8 and CD4 T cells from the subset of IFN-low-response patients 4 d after stimulation (Figures 5D and S9).

In each of these functional assays there was a systematic trend of reduced responses to activating stimuli in lymphocytes from patients with melanoma versus healthy controls. Ten of the comparisons presented in Figure 5 are essentially independent; i.e., the expression of CD69 in each of the four T cells subsets at 6 h; the expression of CD69, CD25, and CD71 in these subsets at 24 h; cytokine expression in T cells; and survival in T cells. The probability of observing this trend across these ten comparisons by chance is very small (*p* < 0.001). These impaired activation responses and reduced survival of CD8 and CD4 T cells from patients with melanoma indicate a distinct immune functional defect in IFN-low-response patients.

## Discussion

Dysfunction or nonresponsiveness of the immune system may be an early event in tumor progression, while global immune suppression develops in most patients with metastatic disease [1,2]. The molecular mechanisms underlying immune dysfunction in cancer remain unclear. In this study, we studied lymphocytes from patients with metastatic melanoma at the level of gene expression using DNA microarrays to identify immune signatures that are associated with the cancer state. To resolve gene expression changes in specific lymphocyte subsets, we analyzed pure cell populations, stringently sorted by flow cytometry to allow precise analysis of each cell type. Our study included all the peripheral blood lymphocyte populations that are potentially involved in antitumor responses and may be negatively impacted by tumors, specifically CD8 T cells, CD4 T cells, B cells, and CD56dim NK cells. Inhibition of these key immune cell subsets in cancer may aid tumor progression and confound immunotherapeutic approaches.

In CD8 T cells, CD4 T cells, and B cells, our results showed that a group of related genes all induced by IFN, e.g., *STAT1, IFIT1,* and *IFI44,* were significantly reduced in expression in patients with melanoma versus healthy controls. These gene expression changes were further validated by qPCR. No statistically significant differences in gene expression were detected in NK cells from patients with melanoma versus healthy controls. The detection of differentially expressed genes that belong to a common pathway validates our approach of using DNA microarrays as a powerful tool to identify pathways and mechanisms of immune dysfunction in cancer. The finding that a group of ISGs is down-regulated in melanoma strongly indicates disruption of IFN-regulated pathways as a dominant mechanism in the dysfunction of immune cells in patients with metastatic melanoma.

IFN-stimulated gene expression is regulated by the JAK-STAT (Janus kinase–signal transduction and activator of transcription) signaling pathway. Binding of type I IFNs, such as IFN-α and IFN-β, to their specific receptor leads to phosphorylation of JAK1 and Tyk2, which in turn phosphorylate STAT2 and STAT1. Phosphorylated STAT1–STAT2 heterodimers associate with IRF9 to form interferon-stimulated gene factor 3 (ISGF3), which binds to interferon-stimulated response elements (ISREs) in the promoters of ISGs, including those observed to be down-regulated in patients with melanoma in our study. Type II IFN (IFN-γ) causes activation and phosphorylation of JAK2 and JAK1, leading to phosphorylation of STAT1. Phosphorylated STAT1 homodimers bind to GAS (γ-activated sequence) elements of ISG promoters [22]. We assessed phosphorylation of STAT1 at tyrosine 701 in response to IFN stimulation, since this is a critical event in transduction of signal from both types of IFN receptors to the nucleus to drive expression of ISGs. Using the Phosflow method we demonstrated that the percentage of lymphocytes from patients with melanoma, particularly T cells, that phosphorylated STAT1 on tyrosine 701, and the mean fold change in intensity of STAT1-pY701 staining in response to IFN-α and IFN-β, were reduced compared to the healthy controls. The defect in STAT1 phosphorylation suggests a mechanism for the low expression of ISGs in T cells from patients with melanoma, since phosphorylation of STAT1 is critical for assembly of ISGF3 for the activation of ISGs. Other studies have documented mutations in components of the JAK-STAT pathway in tumor cells; however, to our knowledge this is the first study to report defects in IFN signaling in immune cells of patients with melanoma.

This defect in lymphocytes from patients with melanoma appears to be specific to type I IFN signaling, since no perturbation of STAT1 phosphorylation was observed in IFN-γ-stimulated samples from patients with melanoma. It is therefore possible that the defect in STAT1 phosphorylation is linked to the function of JAK-STAT components that are unique to the signaling complex induced by type I IFNs, e.g., Tyk2. However, STAT1 phosphorylation at tyrosine 701 is low following IFN-γ stimulation, which may prevent detection of small differences between patient and control samples by Phosflow. Furthermore, there is cross talk between the two classical JAK-STAT signaling pathways induced by type I and II IFNs, and between nonclassical pathways such as the CRKL, p38, and PI3K cascades (reviewed in [23]). Therefore, the reduced phosphorylation of STAT1 in response to IFN-α may also impact IFN-γ signaling in lymphocytes from patients with melanoma in vivo even though such defects are less readily detectable in vitro.

IFNs represent the first line of defense against viral infections and are also involved in immune surveillance against tumors. In the early phases of an immune response, IFNs act as a “third signal” required in addition to the first (antigen) and second (co-stimulation) signals for full activation and memory development rather than tolerance [21]. IFNs support activation and clonal expansion via antiapoptotic and proproliferative effects on both T and B lymphocytes [24–28]. We investigated the functional significance of the observed impaired responses to IFN by assaying the activation and survival of lymphocytes from patients with melanoma. These experiments focused on the subgroup of patients in which low responses to type-I IFNs were observed. This set of IFN-low-response patients showed reduced T cell activation responses and lower survival following stimulation with anti-CD3 and anti-CD28 antibodies. These results demonstrate that lymphocytes from these patients have pronounced functional defects, likely arising from their impaired response to IFN, resulting in lack of the third signal required to initiate the differentiation program for survival and development of effector function and memory. Poor survival and/or increased apoptosis and impaired responses of lymphocytes to stimulation are critical aspects of immune dysfunction in the cancer state. Increased apoptosis has been associated with FasL and other tumor necrosis factor (TNF) family ligands expressed by cancer cells and on tumor-derived microvesicles [4,29–31] and also with tumor-derived gangliosides [32,33]. Reduced activation and clonal expansion of lymphocytes have been linked to disruption of the TCR signaling cascade by decreased expression of CD3 ζ chain and other signaling components [34–37]. Impaired responses to type I IFNs in cancer patients, as shown in our study, represent a novel mechanism by which lymphocytes may be driven into apoptosis and have poor functional responses to activating stimuli in the cancer state. Impaired responses to IFNs at each phase of adaptive immune responses likely contribute to the observed immune low responsiveness and lack of antitumor immunity observed in cancer and may also hinder therapeutic approaches that aim to stimulate antitumor immune responses. The defect in IFN signaling may be directly tumor-induced, or an effect of the general cancer state. Alternatively, the defect may be involved in the development of the melanoma. Our findings may go beyond tumor-induced immune dysfunction; defects in IFN signaling have been documented in other chronic diseases in which immune dysfunction has been described, e.g., chronic hepatitis C infection and multiple sclerosis [38,39].

Phosphorylation of STAT1 is an early proximal event in JAK-STAT signaling; therefore, the defect in STAT1 phosphorylation may be caused by alterations in the function of a limited number of other components, including JAK1, Tyk2, and the regulators of JAK-STAT signaling. The mRNA levels of the IFN receptor subunits STAT2, JAK1, JAK2, and Tyk2 were not significantly different in lymphocytes from patients with melanoma in our microarray data; however, altered function of these molecules may be involved in the impaired phosphorylation of STAT1 in response to IFNs. Regulation of IFN signaling occurs at several levels, including expression and turnover of IFN receptor components, and activation and turnover of intracellular signaling molecules. Three classes of negative regulators have been described to attenuate IFN signaling through their actions on STAT1. These include protein tyrosine phosphatases, such as Src homology 2 (SH2)-containing phosphatase-1 and −2 (SHP-1 and −2) and CD45, protein inhibitors of activated STATS (PIAS) that are constitutively present and function as acute, early regulators of cytokine signaling [40], and the suppressors of cytokine signaling (SOCS) that are rapidly induced by cytokines and form a classical negative feedback loop to regulate JAK-STAT signaling [41]. Tumor or tumor-associated cells actively and directly inhibit immune responses via secretion of immunosuppressive cytokines such as interleukin (IL)-10 [42] and transforming growth factor (TGF)-β1 [43,44], and by skewing CD4 T cell responses to T_H_2 rather than T_H_1 [45,46]. Such cytokines produced in the cancer state may be involved in the mechanism by which IFN signaling is inhibited in lymphocytes in patients with melanoma through their ability to induce expression of the negative regulators of IFN signaling [47–51]. In addition to tumor production of immunosuppressive cytokines, regulatory T cells—which are expanded in cancer patients [52,53]—produce cytokines such as IL-10 and TGF-β1 and may contribute to the inhibition of IFN signaling in lymphocytes in the cancer state.

The low response to IFN-α was partially corrected in lymphocytes from patients with melanoma by prolonged exposure of lymphocytes to high concentrations of IFN-α in vitro, demonstrating that the defect may be reversed by IFN therapy in some patients. There was variation and overlap between healthy and melanoma samples in the Phosflow data and the qPCR data measuring induction of ISGs. This variation indicates that the defect is not significant in all patients with melanoma, but in a subset of patients that specifically rank lowest in their in vitro response to IFN. The reduced response of lymphocytes from some patients with melanoma to high-dose IFN indicates a severe impairment in IFN signaling in a subset of patients. The efficacy of IFN therapy for cancer is thought to be dependent both on direct antiproliferative effects on the tumor and on indirect immunomodulatory effects [54–56]. It is clear from the clinical trials that while some patients respond well to IFN therapy, others are low responders in whom IFN-α has no clinical benefit. A defect in type I IFN signaling in lymphocytes as demonstrated in our study may provide a mechanism for the beneficial effect of IFN in melanoma, and for the resistance to IFN observed in some patients. One of the main limitations of IFN therapy is severe toxicity. Our results may aid in selecting patients likely to have a positive lymphocyte response to IFN and a good clinical outcome, and could avoid unnecessary toxicity in patients with impaired IFN signaling who may be less likely to benefit from high dose IFN therapy.

In summary, we have identified defects in IFN signaling as a dominant mechanism of immune dysfunction in the cancer state. Our study utilized multiple novel technologies to demonstrate a defect in type I IFN signaling in T cells and B cells, but not in NK cells from patients with metastatic melanoma, and that such defects negatively impact the function of these cells. Such lymphocytes also displayed reduced activation and survival in response to in vitro stimulation. The reduced responses to IFNs could be involved in the susceptibility of lymphocytes to spontaneous apoptosis in the cancer state and to general immune nonresponsiveness, both of which are critical aspects of tumor-induced immune dysfunction. Our data also show that the impairment can be partially overcome by prolonged high-dose IFN-α treatment, suggesting a potential mechanism for the efficacy of IFN-α used in the therapy of melanoma. Deregulation of IFN signaling has been described as involved in tumorigenesis [57]; however, to our knowledge this study is the first to find a defect in IFN signaling in immune cells in the cancer state. Our findings represent an important insight into immune dysfunction in cancer, and may lead to novel strategies to correct this dysfunction in cancer patients and to improve immunotherapeutic strategies for cancer.

## Supporting Information

Figure S1 FACS Plots Indicating Sorting Gates for Lymphocyte Subsets Data on PBMCs were acquired and a gate was set on the lymphocytes on a FSC-A versus SSC-A plot (not shown). Gates were set on the CD19^+^ CD3^−^ cells to sort B cells, on CD3^+^ CD56^−^ CD16^−^ CD8^+^ cells to sort CD8 T cells, on CD3^+^ CD56^−^ CD16^−^ CD4^+^ cells to sort CD4 T cells and on CD3^−^ CD19^−^ CD16^+^ CD56dim cells to sort CD56dim NK cells as indicated in the plots.
(1.7 MB TIF)

Figure S2 Microarray Quality Control Plots The vsn function was applied to the raw intensity microarray data after removal of 99 saturated features and one outlier feature.
(A) Microarray data from a dyeswap experiment. Microarrays were hybridized with Cy3-CD8 aRNA and Cy5-TLR (total lymphocyte reference) aRNA, or Cy5-CD8 aRNA and Cy3-TLR aRNA. Cy3-CD8 data from one array and Cy5-CD8 data from the second half of the dye swap experiment were combined, and Cy3 signal was plotted against Cy5 signal.
(B) Microarray data from a self-self experiment. Microarrays were hybridized with Cy3-CD8 targets and Cy5-CD8 targets from the same aRNA sample. Cy3 signal was plotted against Cy5 signal.
(C) Microarray data from replicate samples. Two microarrays were hybridized with Cy3-CD8 and Cy5-TLR targets; Cy3 signal from each array is plotted.
(1.9 MB TIF)

Figure S3 Hierarchical Clustering of Microarray Data This was performed using the 10 ISGs with lowest adjusted *p*-values in B cells (A), CD8 T cells (B), and CD4 T cells (C) from patients with melanoma versus healthy controls. Hierarchical clustering of the microarray data was also performed using the top ranking genes discriminating CD56dim NK cells from patients with melanoma versus controls (D). White indicates highest expression, red indicates lowest gene, and yellow/orange indicates intermediary expression in melanoma versus healthy.
(8.7 MB TIF)

Figure S4 Scatter Plots to Show Percentage of CD8 and CD4 T Cells Expressing CD69 Six Hours after Stimulation Lymphocytes from IFN-low-response patients and healthy donors were stimulated with beads coated with anti-CD3 and anti-CD28 antibodies. Expression of CD69 was measured by flow cytometry 6 h after stimulation in CD8 and CD4 T cells. Healthy (○); melanoma (•).
(4.4 MB TIF).

Figure S5 Correlation of Phosflow data with expression of CD69 in stimulated lymphocytes Lymphocytes from patients with melanoma and healthy controls were stimulated with 1,000 IU/ml IFN-α for 15 min and the percentage of pSTAT1 pY701-positive cells was determined by Phosflow (x-axis). Lymphocytes from the same patients and healthy controls were stimulated with beads coated with anti-CD3 and anti-CD28 antibodies and the percentage of CD69-positive cells was measured by flow cytometry at 6 h (y-axis). (A) Healthy lymphocytes, *r* = −0.0044, *p* = 0.9909; (B) melanoma lymphocytes, *r* = 0.6786, *p* = 0.0643; (C) IFN-low-responder melanoma lymphocytes, *r* = 0.9829, *p* = 0.0027. Correlation coefficients and *p*-values were calculated using the Pearson correlation function in GraphPad Prism v3.02.
(125 KB TIF)

Figure S6 Scatter Plots to Show Percentage of CD8 and CD4 T Cells Expressing CD69 24 Hours after Stimulation Lymphocytes from IFN-low-response patients with melanoma and healthy donors were stimulated with beads coated with anti-CD3 and anti-CD28 antibodies. Expression of CD69 was measured by flow cytometry 24 h following stimulation in CD8 and CD4 T cells. Healthy (○); melanoma (•).
(4.0 MB TIF)

Figure S7 Scatter Plots to Show Percentage of CD8 and CD4 T Cells Expressing CD25 24 Hours after Stimulation Lymphocytes from IFN-low-response melanoma patients and healthy donors were stimulated with beads coated with anti-CD3 and anti-CD28 antibodies. Expression of CD25 was measured by flow cytometry 24 h after stimulation in CD8 and CD4 T cells. Healthy (○); melanoma (•).
(3.7 MB TIF)

Figure S8 Scatter Plots to Show Percentage of CD8 and CD4 T Cells Expressing CD71 Six Hours after Stimulation Lymphocytes from IFN-low-response melanoma patients and healthy donors were stimulated with beads coated with anti-CD3 and anti-CD28 antibodies. Expression of CD71 was measured by flow cytometry 24 h after stimulation in CD8 and CD4 T cells. Healthy (○); melanoma (•).
(2.2 MB TIF)

Figure S9 Scatter Plots to Show Percentage of CD8 and CD4 T Cells Expressing IL-2, TNF-α, and IFN-γ and Percentage Survival of CD8 and CD4 T Cells after Stimulation Lymphocytes from IFN-low-response patients and healthy donors were stimulated with beads coated with anti-CD3 and anti-CD28 antibodies. The percentage of cells expressing IL-2, TNF-α, and IFN-γ was measured 24 h after stimulation. The percentage survival (Annexin V-negative 7-AAD-negative cells) was measured by flow cytometry four days after stimulation. Healthy (○); melanoma (•).
(2.7 MB TIF)

Table S1 Sequences of Primers for Real-Time Quantitative PCR (42 KB DOC)

Table S2 Real-Time Quantitative PCR Analysis Comparing Patients with Melanoma (M) to Healthy Controls (H) for Expression Differences in CD56dim NK Cells of ISGs and Top-Ranking Genes from Microarray Analysis QPCR was used to measure expression of ISGs and top ranked genes shown by microarray analysis to be differentially expressed in melanoma versus healthy samples. The Wilcoxon rank sum test (two-sided) was used to calculate the *p*-values. The estimated log2 fold differences (H/M) between healthy and melanoma with 95% confidence intervals and estimated fold differences (H/M) are shown. The number of healthy and melanoma samples analyzed is indicated.
(40 KB DOC)

### Accession Numbers

The Gene Expression Omnibus (GEO [http://www.ncbi.nlm.nih.gov/geo]) accession number for microarray data from this study is GSE6887. The Entrez Gene accession numbers for the genes discussed in this paper are shown in parentheses: *IFIT3* (3437), *RSAD2* (91543), *LOC129607* (129607), *IFI44L* (10964), *IFIT1* (3434), *IFIT2* (3433), *OAS3* (4940), *FREQ* (23413), *OAS1* (4938), *STAT1* (6772), *IFI44* (10561), *ISG15* (9636), *SAMD9L* (219285), *PARP9* (83666), *CXCL11* (6373), *GBP1* (2633), *CXCL10* (3627), *MX2* (4600), *EIF2AK2* (5610), *LAMP3* (27074), *USP18* (11274), *SAMD9* (54809), *PLSCR1* (5359), *BIRC4BP* (54739), *IFI27* (3429), *MX1* (4599), *HERC5* (51191), CARD9 (64170), CXCL3 (2921), LMO2 (4005), MARCKS (4082), C15orf48 (84419).

## Abbreviations

CI: confidence interval
ISG: interferon-stimulated gene
NK: natural killer
PBMC: peripheral blood mononuclear cell
qPCR: quantitative PCR
TAA: tumor-associated antigen
