# Supplementary material for: Down-Regulation of the Interferon Signaling Pathway in T Lymphocytes from Patients with Metastatic Melanoma
Source: PLoS Med. 2007 May 8;4(5):e176. doi: 10.1371/journal.pmed.0040176 (PMC1865558; doi:10.1371/journal.pmed.0040176)
Supplement: Supplementary file 1 [file pmed.0040176.st001.doc]

| **Entrez Gene Symbol** | **Forward Primer 5'→3'** | **Reverse Primer 5'→3'** |
| --- | --- | --- |
| EIF2AK2 | AGA TTT CTT CGC AAG ACT ATG G | ATC CCG TAG GTC TGT GAA AA |
| IFI44 | CTC TGA GTG GGA GCT GGA CC | TTG CTC AAA AGG CAA ATC CTC T |
| IFIT3 | GGC CCC AAC CTG GGA TTG | CTC GTG ATC CGC CCA CCT |
| IFIT1 | GAT CTG GAA AGC TTG AGC CT | GGG TGC CTA AGG ACC TTG |
| IFIT2 | CTG ACC CAG CAT CAG CCA C | AAT TTT GCA CCC TGA TGA CCT C |
| STAT1 | AGA ATT CGA CAG TAT GAT GAA C | CCT TTC TTT CAT TTC CCT AG |
| OAS1 | TTC AAC ACA GCC CAG GGA TT | AGG CGT GGG TTT CGT GAG |
| MX1 | TGG CCT ATC ACC AGG AGG | AGC ATG GCC TTC TGA AGC |
| MX2 | TGG AAA CCA GCA AAC GTC TC | GCA GCC AGG AAT AGC GAT TT |
| IFI44L | TTT GCT TTT GTT CGT TTT GCC | GGG TGG TTT GCA GTA GGA CC |
| RSAD2 | ATA TGC GCT TTC TGA ACT GTA | AAT CCA GCT TCA GAT CAG C |
| HERC5 | TTG GCT GAC CAG CTT GCT | CCA TGG CTT CAG GCT GC |
| SOD2 | GCC TTA TTC CAC TGC TGG G | TTG CAA GCC ATG TAT CTT TCA G |
| LMO2 | ATC TCA GGC TTT TTG GGC AA | CGC ACT TGA AAC ATT CCA GG |
| CARD9 | TGA GAA CTA CCG CAG GAA | TGT CGG TGT TGT CGC T |
| MARCKS | CCG TTT GTT TGT TGG AGT GG | TTT GAG ATG GGA TCT GTG GG |
| NMES1 | GCT TAT AAC AAT CAA CCA ACA | ACA CTG GTG TCC AGA ATC T |
| FREQ | AGC TGG ATG CCT GGG AA | TGT GTG GAT GCG GAA GAA |
| LAMP3 | TAG CCC CAT CTT GTG GTA ACT T | TGG CAG GCT GCT CTA CAG AA |
| UBC | ATT TGG GTC GCG GTT CTT G | TGC CTT GAC ATT CTC GAT GGT |
