# Supplementary material for: Down-Regulation of the Interferon Signaling Pathway in T Lymphocytes from Patients with Metastatic Melanoma
Source: PLoS Med. 2007 May 8;4(5):e176. doi: 10.1371/journal.pmed.0040176 (PMC1865558; doi:10.1371/journal.pmed.0040176)
Supplement: Supplementary file 2 [file pmed.0040176.st002.doc]

| **Entrez Gene ID** | **EntrezGene Symbol** | **P Value** | **Estimated log2 Fold Differences (Log2(H/M))**  **with 95% C.I.** | **Estimated Fold Differences**  **(H/M)** | **Sample Sizes**  **(H, M)** |
| --- | --- | --- | --- | --- | --- |
| 3434 | IFIT1 | 0.3659 | 1.40, (-1.25, 4.35) | 2.64 | (7, 6) |
| 10561 | IFI44 | 0.5338 | 0.90, (-2.7, 3.2) | 1.87 | (7, 6) |
| 6772 | STAT1 | 0.6806 | 0.40, (-1.2, 2.7) | 1.32 | (7, 9) |
| 5610 | EIF2AK2 | 0.7748 | -0.10, (-1.65, 2.35) | 0.93 | (7, 6) |
| 6648 | SOD2 | 0.1649 | 1.7, (-0.4, 3) | 3.25 | (7, 7) |
| 2921 | CXCL3 | 0.6943 | -0.55, (-3, 1.95) | 0.68 | (8, 7) |
| 4005 | LMO2 | 0.7209 | -0.55, (-2.65, 3.6) | 0.68 | (8, 8) |
| 64170 | CARD9 | 0.7551 | 0.80, (-2.15, 5.2) | 1.74 | (7, 5) |
| 4082 | MARCKS | 0.7922 | 0.43, (-3.25, 5) | 1.34 | (5, 6) |
| 84419 | C15orf48 | 0.8182 | 0.13, (-1.55, 4.35) | 1.09 | (6, 6) |
